# Quantitative Proteomic and Interaction Network Analysis of Cisplatin Resistance in HeLa Cells

**DOI:** 10.1371/journal.pone.0019892

**Published:** 2011-05-26

**Authors:** Juan D. Chavez, Michael R. Hoopmann, Chad R. Weisbrod, Kohji Takara, James E. Bruce

**Affiliations:** 1 Department of Genome Sciences, University of Washington, Seattle, Washington, United States of America; 2 Department of Pharmaceutical Sciences, Himeji Dokkyo University, Himeji, Japan; University of South Florida College of Medicine, United States of America

## Abstract

Cisplatin along with other platinum based drugs are some of the most widely used chemotherapeutic agents. However drug resistance is a major problem for the successful chemotherapeutic treatment of cancer. Current evidence suggests that drug resistance is a multifactorial problem due to changes in the expression levels and activity of a wide number of proteins. A majority of the studies to date have quantified mRNA levels between drug resistant and drug sensitive cell lines. Unfortunately mRNA levels do not always correlate with protein expression levels due to post-transcriptional changes in protein abundance. Therefore global quantitative proteomics screens are needed to identify the protein targets that are differentially expressed in drug resistant cell lines. Here we employ a quantitative proteomics technique using stable isotope labeling with amino acids in cell culture (SILAC) coupled with mass spectrometry to quantify changes in protein levels between cisplatin resistant (HeLa/CDDP) and sensitive HeLa cells in an unbiased fashion. A total of 856 proteins were identified and quantified, with 374 displaying significantly altered expression levels between the cell lines. Expression level data was then integrated with a network of protein-protein interactions, and biological pathways to obtain a systems level view of proteome changes which occur with cisplatin resistance. Several of these proteins have been previously implicated in resistance towards platinum-based and other drugs, while many represent new potential markers or therapeutic targets.

## Introduction

Many current cancer chemotherapy strategies involve disruption of tumor cell growth by interfering with mitosis or by causing cancer cells to commit to apoptotic pathways. Cisplatin is a powerful chemotherapeutic cytotoxin primarily targeting DNA to form DNA cross-links that can halt cell replication and can activate a series of signal transduction pathways ultimately leading to cell death. Acquired and intrinsic resistance to cisplatin continues to be a major problem for successful clinical treatment [1]. Generally, acquired resistance is the most common reason for cancer chemotherapy failure. Possible mechanisms for resistance to cisplatin include reduced intracellular concentration of cisplatin by increased drug efflux and/or decreased drug influx, increased inactivation by reaction with glutathione and other intracellular nucleophiles, increased repair of DNA damage, and altered apoptotic signaling pathways [1], [2]. Cisplatin along with other platinum based drugs such as carboplatin and oxiplatin, are seeing a resurgence of clinical use in combination with other cytotoxic compounds to treat various carcinomas including ovarian, colorectal, prostate, lung and breast cancer [3].

Tissue culture studies on drug resistant cell lines generated by continuous exposure of parental cells to chemotherapeutic agents have generated much of the current knowledge on the mechanisms of drug resistance. Genomic microarray experiments are the most commonly applied to study drug resistance and have successfully identified many genes with altered expression levels in drug resistant cell lines [4], [5]. However the interpretation of purely transcriptomic data is limited in its ability to provide a systems level understanding of changes to the proteome. The proteome is a much more dynamic environment than the transcriptome due to the kinetics of protein turnover, post-translational modifications and protein-protein interaction networks Several studies suggest that quantitative mRNA measurements do not always reflect protein expression levels, likely due to post-transcriptional changes in protein abundances [6], [7], [8], [9]. Comparative proteomics studies have also been applied to the problem of drug resistance in cancer [10]. The majority of these studies have relied on traditional proteomics techniques based on quantitative densitometry analysis using two dimensional gel electrophoresis, or Western blotting along with protein identification by peptide mass fingerprinting. While these methods generally provide a robust means of profiling changes in protein expression patterns, identification is labor intensive requiring that each protein spot of interest be excised and processed separately. Quantification in gel-based methods is also challenging due to many factors including overlapping spots, weak signal intensity, spot positional differences, and mismatched protein spots.

Proteins function as the mediators of nearly all cellular processes, either directly or through interactions with other biomolecules comprising what has been termed the interactome [11]. Quantitative proteomic expression level data will serve as an important part of understanding the complex molecular networks which underlie the emergence of drug resistance. Knowledge of altered protein expression levels and perturbations to the interactome will assist in the development of targeted-network combination therapies with the potential to overcome drug resistance [12]. However quantitative proteomics data will need to be integrated with functional biochemical information to reveal the actual mechanism of drug resistance.

In the present study we have applied an analytical quantitative proteomics approach employing stable isotope labeling with amino acids in cell culture (SILAC), to study changes to the proteome with acquired drug resistance to cisplatin in the HeLa cervical carcinoma cell line. The cisplatin resistant cell line HeLa/CDDP was generated previously under exposure to a clinically relevant concentration of cisplatin (1 µM) and was found to display a 2.6 fold increase in resistance, compared to non-resistant HeLa cells, to cisplatin and similar increase in resistance towards other platinum based drugs [13]. Here we identify and quantify a total of 856 proteins with 374 proteins displaying significantly altered abundance levels between the cisplatin resistant and sensitive cells.

## Results

### Identification of proteins differentially expressed between cisplatin resistant and sensitive HeLa cells

**Figure 1:**
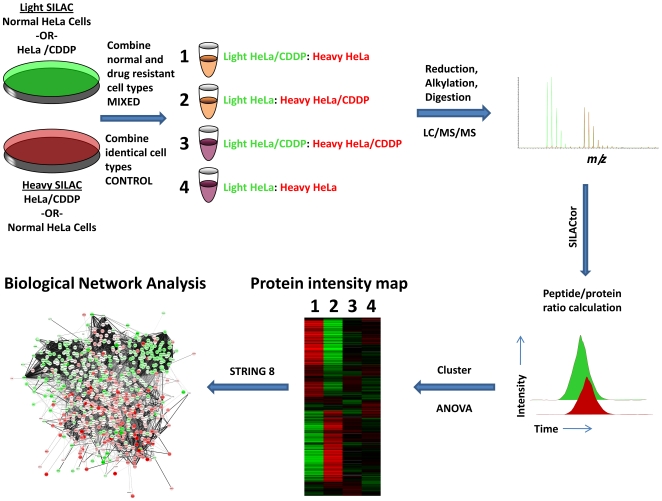
Overview of experimental design. HeLa and HeLa/CDDP cells were grown on isotopically light or heavy media. Light and heavy cell lysates from HeLa and HeLa/CDDP were combined to generate forward and reverse mixed SILAC samples. Light and heavy cell lysates of the same cell type were combined to generate control samples. Protein samples were reduced, alkylated and digested with trypsin into peptide samples that were then analyzed by LC-MS/MS. Quantification of SILAC peptide pairs was performed with the software tool, SILACtor. A heat map was used to visualize proteins displaying increased and decreased expression between HeLa and Hela/CDDP. Finally biological network analysis was combined with protein expression level data to identify biological pathways relevant to cisplatin resistance.

Proteomics analysis using SILAC technology was employed to quantify protein expression level differences between cisplatin resistant and cisplatin sensitive HeLa cells. A schematic illustrating the experimental outline is shown in **Figure 1**. In total we were able to identify and quantify 1232 proteins, of which 856 were observed in at least 50% (3/6) of the mixed SILAC samples (HeLa/CDDP:HeLa mixed at a 1∶1 ratio) samples and 50% (3/6) of the control samples (HeLa/CDDP or HeLa mixed at a 1∶1 ratio with themselves). The average false discovery rate (FDR) for peptide identification was 0.87%, determined by searching a randomized decoy database with Mascot. **Table S1** displays the full list of the 856 proteins along with their SILAC ratios.

**Figure 2:**
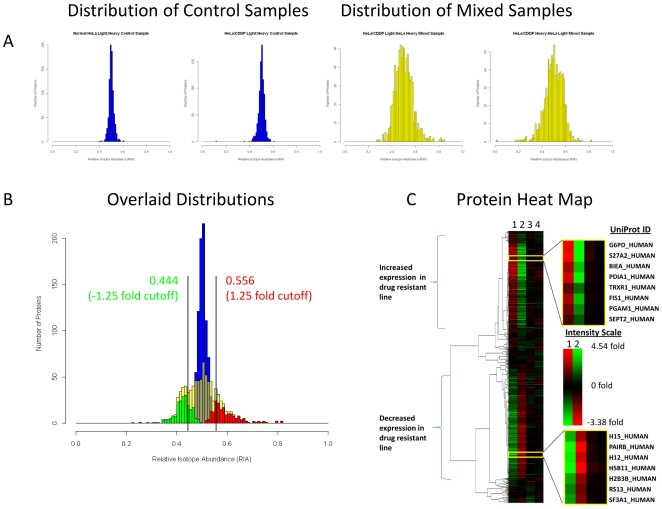
Distribution and heat map analysis of quantified proteins. **A** Histograms illustrating of the distribution of RIA’s observed for the control samples (blue) and the mixed samples (yellow). **B** Histogram of the distribution of averaged RIA’s with overlaid distributions of the proteins identified by ANOVA with significantly decreased ratios (green) and significantly increased ratios (red). Bars are included indicating a±1.25 fold change cutoff which was determined from cumulative distribution analysis of the control sample at the 99% confidence level. **C** Heat map generated from SILAC data comparing cisplatin resistant HeLa cells to normal HeLa cells. Lane **1** is light cisplatin resistant and heavy normal cells. Lane **2** is heavy cisplatin resistant and light normal cells. Lane **3** is a one to one mixture of light and heavy cisplatin resistant cells. Lane **4** is a one to one mixture of light and heavy normal cells.

Histograms illustrating the distribution of relative isotope abundance (RIA, area of light isotopic distribution divided by the sum of the areas for the light and heavy isotopic distributions) values for the control samples and the mixed samples are shown in **Figure 2A**. The protein ratios from the mixed samples show a broad distribution compared to those from the control samples indicating that several proteins are differentially expressed in HeLa/CDDP versus the drug sensitive cells. To determine which proteins showed altered expression in HeLa/CDDP compared to HeLa, an analysis of variance (ANOVA) was performed to compare the mean SILAC ratio for each protein from the mixed sample type to the control sample type. The resulting F-statistic and corresponding p-values from the ANOVA are included in **Table S1.** In total 387 proteins were found to have significantly shifted expression levels (p<0.01), of which 184 proteins were observed with increased expression levels and 203 proteins were observed with decreased expression levels in HeLa/CDDP. The 184 proteins with increased expression levels had measured RIA ratios between 0.516 and 0.819 with an average of 0.595, corresponding to a range from 1.07 to 4.54 fold increase with an average of 1.54 fold increase in expression levels. The 203 proteins with decreased expression levels had measured RIA ratios between 0.490 and 0.228 with an average of 0.422, corresponding to a range from 1.04 to 3.38 fold change with an average of 1.39 fold decrease in expression levels. **Table 1** lists the ten proteins with the largest increase in expression levels and the ten proteins with the largest decrease in expression levels. **Figure 2B** displays the distributions of RIA values for the proteins identified by ANOVA with significantly altered expression levels. To further examine the magnitude of change in protein expression levels that could be expected to result from normal biological variation, cumulative distribution analysis was performed on the control samples, resulting in that a 1.25 fold change or greater (RIA less than 0.444 or RIA greater than 0.556) is not due to biological noise at the 99% confidence level. A total of 142 proteins from the 184 proteins with increased expression and with ANOVA p-values <0.01 also exceed the 1.25 fold change cutoff, while a total of 152 proteins from the 203 proteins with decreased expression and p-values <0.01 exceed the −1.25 fold change cutoff. The magnitude of the observed protein expression level changes is on the same order as the increased relative resistance of HeLa/CDDP vs. HeLa to cisplatin (2.6 fold) [13]. Hierarchical clustering of the 856 proteins based on their SILAC ratios was performed to generate a dendrogram and colored heat map shown in **Figure 2C**. The heat map contains four columns corresponding to the two mixed SILAC samples and two control SILAC samples (column 1-light HeLa/CDDP:heavy HeLa, column 2-heavy HeLa/CDDP:light HeLa, column 3- light HeLa/CDDP:heavy HeLa/CDDP, column 4- light HeLa:heavy HeLa). The heat map image reveals three general clustered regions which include proteins with increased expression in HeLa/CDDP, proteins with decreased expression in HeLa/CDDP, and proteins with unchanged expression levels. Each lane in the map is an average of three biological replicates each consisting of technical duplicates. In general excellent agreement is observed between the inverse mixed SILAC samples (**Figure 2C** **, columns 1 & 2**). A heat map displaying the signals from each of the biological replicates is included as **Figure S1**.

**Table 1:** Proteins with largest increased and decreased expression levels in HeLa/CDDP.

Ten Proteins With Largest Increase in Expression Levels in HeLa/CDDP				
UniProt ID	Protein Name	Change*	p-value†	GO Biological Pathway‡
ALDR_HUMAN	Aldose reductase	4.54±0.20	1.73E-03	carbohydrate metabolic process
CATD_HUMAN	Cathepsin D	4.40±0.03	2.64E-05	autophagic vacuole assembly
G6PD_HUMAN	Glucose-6-phosphate 1-dehydrogenase	3.93±0.03	1.26E-08	NADPH regeneration
S10A4_HUMAN	Protein S100-A4	3.78±0.03	1.01E-05	positive regulation of I-kappaB kinase/NF-kappaB cascade
1C05_HUMAN	HLA class I histocompatibility antigen, Cw-5 alpha chain	3.16±0.06	1.93E-03	immune response
NQO1_HUMAN	NAD(P)H dehydrogenase [quinone] 1	2.89±0.04	6.50E-07	response to oxidative stress
IDHP_HUMAN	Isocitrate dehydrogenase [NADP], mitochondrial	2.86±0.03	8.19E-04	2-oxoglutarate metabolic process
CHSP1_HUMAN	Calcium-regulated heat stable protein 1	2.83±0.02	2.89E-08	intracellular signaling pathway
VAT1_HUMAN	Synaptic vesicle membrane protein VAT-1 homolog	2.69±0.02	2.74E-04	oxidation reduction
APT_HUMAN	Adenine phosphoribosyltransferase	2.66±0.03	7.43E-08	adenine metabolic process

Ten Proteins With Largest Decrease in Expression Levels in HeLa/CDDP				
UniProt ID	Protein Name	Change*	p-value†	GO Biological Pathway‡
H12_HUMAN	Histone H1.2	−1.85±0.05	1.01E-05	nucleosome assembly
NC2A_HUMAN	Dr1-associated corepressor	−1.91±0.05	7.98E-03	negative regulation of transcription from RNA polymerase II promoter
PGM1_HUMAN	Phosphoglucomutase-1	−1.92±0.03	6.91E-08	glucose metabolic process
RL1D1_HUMAN	Ribosomal L1 domain-containing protein 1	−1.92±0.14	2.49E-02	RNA processing
BRX1_HUMAN	Ribosome biogenesis protein BRX1 homolog	−1.93±0.02	2.10E-06	ribosome biogenesis
GNAI3_HUMAN	Guanine nucleotide-binding protein G(k) subunit alpha	−2.11±0.04	1.43E-04	negative regulation of adenylate cyclase activity
EBP2_HUMAN	Probable rRNA-processing protein EBP2	−2.22±0.06	2.06E-03	ribosome biogenesis
PAIRB_HUMAN	Plasminogen activator inhibitor 1 RNA-binding protein	−2.29±0.05	9.36E-07	regulation of anti-apoptosis
GBG12_HUMAN	Guanine nucleotide-binding protein G(I)/G(S)/G(O) subunit gamma-12	−2.50±0.05	5.72E-05	cerebral cortex development
HSB11_HUMAN	Heat shock protein beta-11	−3.38±0.07	1.05E-04	cell adhesion

List of 20 proteins including the ten proteins with largest increased expression levels and ten proteins with the largest decreased expression levels between the cisplatin resistant and sensitive cells. A complete list with the measured expression levels of the 856 proteins can be seen in **Table S1.**

*Fold change of protein level measured by SILAC (-) denotes a fold decrease ± standard deviation.

† p-value determined from ANOVA (p<0.01) considered significant.

‡ First biological pathway Gene Ontology term associated with protein.

### Western Blot

**Figure 3:**
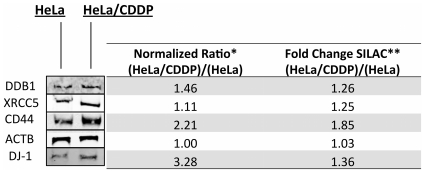
Western blot analysis. Cropped image of Western blot results comparing levels of four proteins (DDB1, Ku80, CD44, DJ-1) between HeLa and HeLa/CDDP. ACTB was also monitored as a quantitative control. * The normalized ratio for each protein was calculated by averaging the signal intensity from three biological replicates from HeLa/CDDP and dividing it by the average signal intensity from 3 biological replicates from HeLa. This ratio was then normalized using the average ratio of signal intensity of ACTB to the respective protein of interest band. In general there is good agreement between the ratios obtained from Western blotting and from SILAC. Full Western blot images are available in **Figure S2**.

To confirm the differential expression of a few key target proteins including CD44, DDB-1, DJ-1 and XRCC5, Western blotting was performed. Levels of β-actin were monitored as a quantitative control. A cropped version of the Western blot results with a comparison of the protein ratio calculated from the Western blot and the SILAC results can be seen in **Figure 3**. In general there is good agreement between the two techniques as each of these proteins was identified with increased expression levels in HeLa/CDDP compared with normal HeLa. Images of the full Western blots for each of the selected proteins are included in **Figure S2**.

### Biological Pathway Analysis of Differentially Expressed Proteins

**Figure 4:**
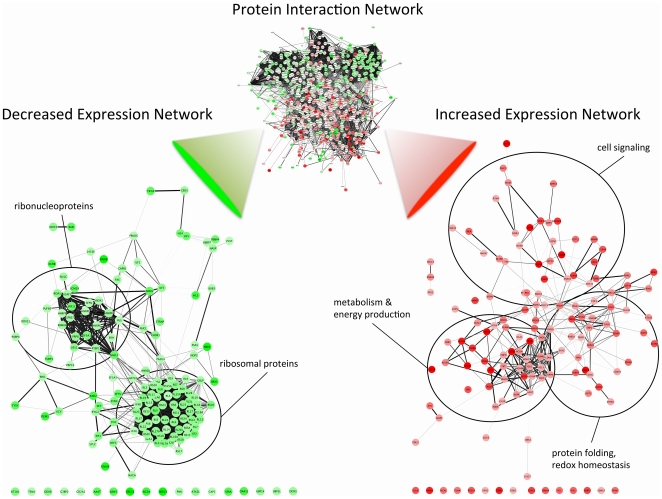
Expression level and protein interaction network analysis. Protein interaction network generated with STRING 8.3 [56] and visualized with Cytoscape [57] consisting of 803 proteins connected by 1174 protein-protein interactions. The 134 proteins with significantly increased expression levels (p<0.01) of at least 1.25 fold in cisplatin resistant HeLa cells are shown in red. Major clusters of interacting proteins include those involved in metabolic energy production, protein folding, and cellular signaling. The 147 proteins with significantly decreased expression levels (p<0.01) of at least 1.25 fold in cisplatin resistant HeLa cells are shown in green. Major clusters of interacting proteins include the 40S and 60S ribosomal proteins and ribonucleoproteins. Interactive Cytoscape networks are included as **Dataset S1**.

The protein interaction network shown in **Figure 4** contains 803 proteins and 1174 protein-protein interactions. An interactive version of this Cytoscape network is provided in **Dataset S1**. Several clusters of related functional classes of proteins are visible in this network including ribosomal proteins, ribonucleoproteins, metabolic and energy producing proteins, and proteins involved in redox homeostasis and protein folding. To identify the relevant biological pathways that were altered due to cisplatin resistance, BiNGO [14] was used to find GO biological pathway and molecular function terms that were enriched among the differentially expressed proteins in the network. In total 208 biological pathway terms and 109 molecular function terms were associated with proteins identified with increased expression, while 101 biological pathway terms and 18 molecular function terms were associated with proteins identified with decreased expression. Enriched biological pathways for proteins identified with increased expression include the metabolism of carbohydrates, metabolism of NADH and NADPH, regulation of apoptosis, protein folding, and maintenance of cellular homeostasis. Enriched biological pathways for proteins identified with decreased expression include ribosomal assembly and RNA processing, gene expression, and translation. A complete list of the enriched GO biological pathway and molecular function terms for the proteins with altered expression levels can be viewed in **Table S2.**

## Discussion

The combination of quantitative mass spectrometry and biological network analytical tools allow for a systems-level view of changes to the proteome that are associated with chemoresistance. Relative expression levels of proteins that carry out key molecular functions in biological pathways associated with cisplatin resistance were mapped.

### Increased glycolysis and energy producing metabolic pathways

**Figure 5:**
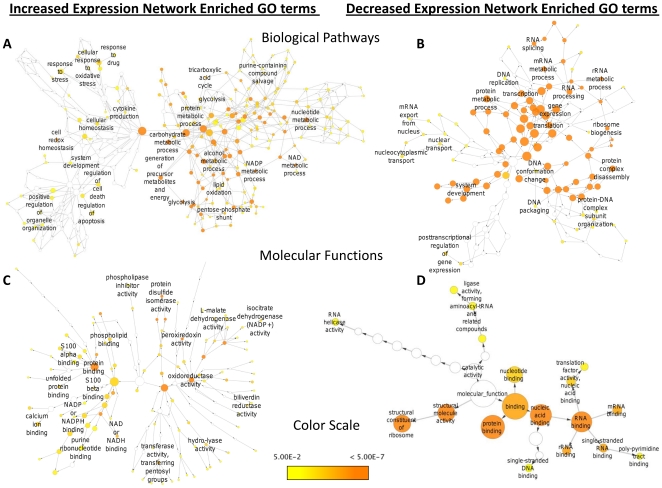
Biological pathway and molecular function networks. BiNGO [14] generated biological pathway and molecular function networks for proteins with significantly altered expression levels associated with cisplatin resistance. Node size is related to the number of proteins associated with a GO term, while the color relates to the p-value for the statistical significance of the enrichment of a GO term**.** Interactive Cytoscape networks are included **as Dataset S1**.

Several enriched biological pathways from the subset of proteins identified with increased expression in HeLa/CDDP are related to glycolysis and carbohydrate metabolism (**Figure 5**). Thirty proteins involved in carbohydrate metabolism, including ten glycolytic and four pentose phosphate pathway enzymes were measured with increased expression in HeLa/CDDP (**Table S2**). Increased glycolytic activity under normal aerobic conditions, referred to as “the Warburg effect”, is a common feature observed in many malignant cancers and is associated with conditions including hypoxia, acidosis, and mitochondrial dysfunction [15]. These conditions can lead to enhanced chemoresistance and malignancy through activation of the hypoxia-induced factor (HIF) system [15], [16]. It was recently shown that HIF-1α confers chemoresistance by modulating p53 and nuclear factor-κB (NF-κB) activity [17]. Due to these observations inhibition of glycolysis is emerging as a potential therapeutic approach for overcoming drug resistance in cancer [15], [18]. Additional enriched metabolic pathways with enzymes displaying increased expression levels in HeLa/CDDP include the TCA cycle, lipid metabolism and NAD^+^/NADP^+^ metabolic processes (**Figure 5** **,** **Table S2**). Elevated production of NADH and NADPH by HeLa/CDDP would help compensate for the cellular stress and generation of reactive oxygen species (ROS) due to DNA and protein damage caused by cisplatin treatment. Aldose reductase (ALDR) had the highest increased expression levels (4.54 fold) in HeLa/CDDP of any of the proteins identified in this study (**Table 1**). Several direct protein interaction partners of ALDR also had significantly increased levels including glucose-6-phosphate 1-dehydrogenase (3.93 fold), alpha-aminoadipic semialdehyde dehydrogenase (2.15 fold), transaldolase (1.82 fold), transketolase (1.40 fold), and carbonyl reductase (1.39 fold) suggesting the related functions and pathways of these enzymes play an important role in cisplatin resistance. ALDR catalyzes NADPH-dependent reduction of a wide range of aldehyde containing compounds to their corresponding alcohols, participating in the polyol pathway and oxidative stress response. Inhibition of ALDR activity was shown to enhance chemotherapy sensitivity in HeLa cells through activation of the extracellular signal-related kinases (ERK) pathway [19]. Additionally, clinical studies have shown both overexpression and increased activity of ALDR exist in a number of human cancer tissues [20].

### Redox homeostasis and stress response related proteins

*In vivo* cisplatin becomes aquated and acts as a potent electrophile, covalently modifying nucleophilic sites on proteins, DNA and RNA. The cellular damage caused by cisplatin results in oxidative stress and formation of ROS, which are important in the apoptotic mechanism of cisplatin, but have also been implicated in its nephrotoxic side effects [21], [22]. We observed several enriched pathways and molecular functions related to redox homeostasis and stress response to be associated with proteins displaying increased expression in HeLa/CDDP (**Figure 5** **,** **Table S2**). Increased levels of glutathione (GSH) and proteins in the GSH system are frequently associated with cisplatin resistance [2], [9], [13]. Glutathione related enzymes including glutathione transferases (GSTs) kappa 1 (GSTK1) and omega 1 (GSTO1) were detected with 1.82 and 1.48 fold increased expression levels respectively in HeLa/CDDP. Elevated levels of GSTs are generally associated with anticancer drug resistance, and have emerged as promising therapeutic targets [23]. In addition to catalyzing the conjugation of GSH to electrophilic compounds, GSTs also increase resistance to apoptosis by inhibiting the mitogen activated protein kinase (MAPK) pathway. Results from a recent study demonstrated that the overexpression of GSTO1 in HeLa cells confers resistance to cisplatin primarily through the activation of the phosphatidylinositol-3 kinase/serine/threonine kinase (PI3-K/AKT) pathway and inhibition of the c-Jun N-terminal kinases (JNK) apoptotic pathway [24]. We also observed elevated levels of peroxiredoxins, which are involved in pathways of stress response and regulation of apoptosis, associated with increased levels in HeLa/CDDP (**Figure 5** **,** **Table S2**). Peroxiredoxins are antioxidant enzymes that decompose peroxides and are functionally recycled by glutathione and GST enzymes. We identified peroxiredoxins 2, 4, 5 and 6 with increased expression levels (1.30, 1.51, 1.60, and 1.29 fold increases respectively) in cisplatin resistant cells. Overexpression of peroxiredoxins 2 and 6 have been shown to confer resistance to cisplatin by lowering ROS levels and inhibiting caspase signaling pathways [25], [26].

We also observed several chaperone proteins with significantly increased expression levels in the cisplatin resistant cells including, protein disulfide isomerases (PDIA1, PDIA3, PDIA4, PDIA6), 10 kDa heat shock protein, calnexin, peptidyl-prolyl cis-trans isomerase A, calreticulin, hypoxia up regulated protein 1, endoplasmin, prefoldin subunit 3, and protein DJ-1. In contrast, heat shock protein beta-11 was identified with the most decreased expression levels (−3.38 fold) in HeLa/CDDP (**Table 1**).

### Signaling proteins

Protein DJ-1, is an oncogene transcription factor which also functions in cellular transformation and oxidative stress response. DJ-1 was identified with increased expression (1.36 fold by SILAC, 3.28 fold by Western blot) in HeLa/CDDP **Figure 3**. Overexpression of protein DJ-1 has been shown to increase cellular survival by inhibition of JNK pathway and by acting as a negative regulator of the tumor suppressor phosphatase and tensin homolog leading to activation of the PI3-K/AKT pathway [27]. Another study demonstrated DJ-1 exerts cytoprotective action by inhibiting Bcl-2–associated X protein (Bax) transcription by p53 [28].

CD44 is an outer membrane protein which acts as a receptor for the glycosaminoglycan hyaluronan and was identified with significantly increased levels (1.85 fold by SILAC, 2.21 fold by Western blot,) in the cisplatin resistant cells (**Figure 3**). Hyaluronan and CD44 have been shown to influence drug resistance through several mechanisms including cell survival signaling pathways, drug transporter expression and activity, glycolytic phenotype, and cancer stem-like cell characteristics [29]. Hyaluronan was found to promote CD44 dependent cisplatin resistance in head and neck cancer [30]. The interaction between hyaluronan and CD44 has been shown to regulate PI3-K/AKT signaling and downstream anti-apoptotic events [31]. CD44 is also one of the most common markers for isolating cancer stem-like cells, which are a highly malignant subpopulation of cells that display drug and radiation resistance and have been characterized in many cancers [29]. Because of these properties it has emerged as a promising therapeutic target for mitigating the effects of drug resistance and malignancy in cancer and is currently a prime therapeutic target for glioblastoma [32]. As Xu et al. show, upregulation of CD44 may constitute a key event in development of cancer cell resistance to cellular stresses of a variety of different origins. CD44 is also a known binding partner of ezrin/radixin/moesin (ERM) proteins which function to crosslink actin filaments with plasma membranes [33]. Moesin was identified with 1.41 fold increased levels in HeLa/CDDP, suggesting that CD44 and its interacting partner moesin both play an important role in cisplatin resistance.

The S100 family of proteins consists of small Ca^2+^-binding EF-hand proteins that are known to play a diverse set of roles in many cancers [34]. In our study S100-P, S100-A4, S100-A6, S100-A11 and S100-A13 were all identified with increased levels (2.48, 3.78, 1.71, 1.86, 1.74 fold increases respectively) associated with cisplatin resistance. Enriched S100α and S100β binding were identified in the increased expression GO molecular function network in **Figure 5**. S100-A4 and S100-A6 have attracted significant attention because of their established significance in the progression of metastatic tumors [35]. Evidence shows S100-A4 inhibits phosphorylation of p53, thereby interfering with its transcriptional activity and reducing p53 induced apoptosis [36]. Increased gene expression levels of S100-A4 are known to promote metastasis and have been linked with chemoresistance in pancreatic and colon cancer cell lines [37], [38]. The aspartic protease, cathepsin D (CATD), was identified with the second largest increase in expression levels (4.40 fold) **Table 1**. CATD has emerged as a potential therapeutic target in cancer as it is an important regulator of apoptosis, and promotes invasive and metastatic properties in tumors [39]. Interestingly CATD has direct connections with S100-A4, GSTO1, and CD44 in our protein interaction network (**Figure 4**), suggesting these interactions could play an important role in apoptotic control in HeLa/CDDP.

Annexins are another large family of Ca^2+^-binding proteins, fulfilling a wide range of cellular functions including vesicle trafficking, cell division, apoptosis, and calcium signaling. Several annexins are also known to interact and form complexes with S100 proteins (e.g. annexin 1 with S100A11, annexin 2 with S100A10, and S100A4, and annexin 4 and annexin 11 with S100A6) [40]. Annexins A2, A4, A5, A6, A7 and A11 were all detected with increased expression levels in cisplatin resistant HeLa cells. Increased levels of annexin A4 have been associated with cisplatin resistance in ovarian cancer and paclitaxel resistance in the lung cancer cell line H460 [9], [41]. Clearly the annexin and S100 protein families play important roles in cancer biology and drug resistance making them interesting targets for future studies.

Enrichment of the heme catabolic pathway is observed in **Figure 5** and **Table S2,** due to increased expression of both isozymes of biliverdin reductase (biliverdin reductase A, BIEA; and B, BLVRB) (1.67 and 1.46 fold increase respectively) in HeLa/CDDP. Overexpression of biliverdin reductase enhances resistance to multiple drugs including cisplatin and doxorubicin, possibly by mediating protein kinase C activity; however the exact mechanism of enhanced resistance is unclear [42]. The enzymatic product, bilirubin is a potent antioxidant and has been shown to induce expression of multi-drug resistance associated protein and resistance to cisplatin when added to cultured cells in a conjugated form [43].

### DNA binding and damage repair proteins

While the exact molecular mode of action of cisplatin is not completely understood, it is clear that DNA is its primary target. Therefore increased activity of DNA repair mechanisms are implicated in the resistance to cisplatin [2]. We identified DNA damage-binding protein 1 (DDB1) and X-ray repair cross-complementing protein 5 (XRCC5) with significantly increased expression levels (1.26 and 1.25 fold increases respectively) in the cisplatin resistant cells. The expression levels of DDB1 and XRCC5 were also measured by Western blot to be 1.46 fold and 1.11 higher in HeLa/CDDP respectively (**Figure 3**). However, no enriched DNA repair pathways were identified indicating that peripheral functions of these proteins could be important in the mechanism of cisplatin resistance. In addition to participating in nucleotide excision repair, DDB1 has been shown to regulate the cell cycle under conditions of genotoxic stress by targeting the protein kinase Chk1 to the Cul4 E3-ubiquitin ligase [44]. Similarly, XRCC5 participates in non-homologous end joining but also suppresses p53-dependent DNA damage response [45]. Interestingly, we identified the DNA-dependent protein kinase catalytic subunit (PRKDC) with 1.27 fold decreased expression levels in HeLa/CDDP. PRKDC along with XRCC5 and XRCC6 form the DNA-dependent protein kinase complex (DNA-PK) which is activated in response to DNA damage and participates in non-homologous end joining and V(D)J recombination. Traditionally it has been thought that Ku (a heterodimer of XRCC5 and XRCC6) first binds to the site of DNA damage and recruits PRKDC to the damaged site to initiate repair. However it has been shown that PRKDC can be activated in the absence of Ku [46]. In response to DNA damage, PRKDC has been shown to be an upstream regulator of p53 induced apoptosis [47]. These results indicate that lower levels of PRKDC may result in decreased p53 induced apoptosis through the PRKDC regulated pathway in HeLa/CDDP.

### Ribosomal proteins

In our study we detected several ribosomal proteins including 32 proteins of the 60 s ribosomal subunit and 20 proteins of the 40 s ribosomal subunit, all of which had significantly decreased levels in the cisplatin resistant cells. Similar results were observed in a genome microarray study of cisplatin resistant esophageal cancer cells that found a relatively large number of underexpressed ribosomal genes to be associated with cisplatin resistance [5]. A large cluster of ribosomal proteins can be seen in the protein interaction network in **Figure 4B**. The consistent under expression of the ribosomal proteins is interesting in light of evidence that cisplatin inhibits ribosomal RNA synthesis and assembly of the ribosomal complex [48], [49]. It’s also important to consider many ribosomal proteins have peripheral functions related to apoptosis, DNA repair and oncogenesis [5], [50]. Therefore decreased levels of these proteins are likely important factors in the mechanism of cisplatin resistance. Additionally deregulation of ribosomal transcription could exacerbate the disparity between mRNA and protein expression levels, which has been observed with cisplatin resistant cells [9].

### Concluding comments

In summary, it is clear that the molecular mechanisms contributing to cisplatin resistance comprise a complex multifactorial network. This study demonstrates that quantitative proteomics analysis using SILAC is a useful technique to identify key molecular changes which occur with acquired resistance to cisplatin. Using SILAC we were able to monitor expression levels for 856 proteins and identify 374 proteins with statistically significant altered expression levels. Importantly, in contrast to many drug resistant cell line based studies, these differences were measured on a drug resistant cell line developed under clinically relevant levels of cisplatin. These results corroborate known mechanisms associated with cisplatin resistance and provide new details on the molecular players involved.

Drug resistance remains a major hurdle for the successful treatment of cancer. Quantitative proteomic studies such the one here, are able to identify potential protein targets and cellular pathways that are altered in the drug resistant phenotype. However, this type of data really only provides a single snapshot view of the complete cellular processes involved. It is also important to consider that in shotgun proteomics experiments such as the one here, peptides are identified in a data dependent fashion which results in a bias towards more abundant proteins and therefore not all proteins in the cell will be measured. However, as our results show, there are significant differences in protein expression levels between the cisplatin sensitive and resistant cells even among the proteins we were able to measure. In future studies, selected reaction monitoring (SRM) methods can be used to extend quantitative proteomics studies on drug-resistant cell lines to proteins with lower expression levels. Ultimately, integration of protein expression profiling information with studies probing changes to the dynamics of the proteome and protein-protein interactions are needed to obtain a more complete view of perturbations to the interactome. Incorporating interactome data into the broader context of cellular functional pathways will help lead to a mechanistic understanding of drug resistance. New therapeutic strategies based upon such knowledge hold the promise of individualized treatment with a higher probability of success.

## Materials and Methods

### Chemicals

L-arginine-^13^C_6_ and L-lysine-^13^C_6_^15^N_2_ were obtained from Cambridge Isotope Laboratories (Andover, MA). Dialyzed fetal bovine serum (FBS) was purchased from PAA Laboratories (Etobicoke, Ontario). Hyclone® penicillin-streptomycin 100x solution and Pierce Coomassie Plus reagent were purchased from Thermo Fisher Scientific (Waltham, MA). Antibodies for CD44, DDB-1, DJ-1 and Ku80 (XRCC5) were purchased from Cell Signaling Technology (Danvers, MA). Anti-β-actin antibody was purchased from Sigma (St. Louis, MO). Goat anti-rabbit IRDye 680 and goat anti-mouse IRDye 800 antibodies were supplied by LI-COR Biosciences (Lincoln, NE). Modified trypsin was purchased from Promega (Madison, WI). Dulbecco’s Modified Eagles Medium (DMEM) was custom prepared according to the formulation by Invitrogen (http://www.invitrogen.com/site/us/en/home/support/Product-Technical-Resources/media_formulation.45.html). All other chemicals were reagent or molecular biology grade and were purchased from Sigma (St. Louis, MO).

### Cell Culture

HeLa cells [13] were cultured in DMEM supplemented with 10% FBS and 100 units/mL penicillin-streptomycin, in a humidified atmosphere containing 5% CO_2_ at 37°C with media renewal every 2–3 days. The cisplatin resistant derivative line, HeLa/CDDP [13], was maintained in identical conditions and media with the addition of 1 µM cisplatin. For SILAC experiments, cells were cultured in heavy DMEM containing L-arginine-^13^C_6_ and L-lysine-^13^C_6_^15^N_2_ for at least five cell doublings. Cells were harvested when they reached 80% confluency by aspirating off the media, washing with ice cold 100 mM NH_4_HCO_3_, then scraped off the dishes and stored at −80°C.

### Sample Preparation

Cells were ruptured by repeated rapid freeze/thaw cycles using liquid N_2_ and an ultrasonic water bath. The total protein concentration was determined with the Bradford protein assay. SILAC samples were prepared by mixing equal amounts of light or heavy protein from normal HeLa cells with corresponding heavy or light protein from HeLa/CDDP cells. Control samples were prepared by mixing equal amounts of light and heavy protein from the same cell type. Disulfide bonds were reduced with 5 mM TCEP for 30 minutes, followed by alkylation with 10 mM IAA for 30 min in the dark at room temperature. Protein samples were digested with a 1∶200 ratio of trypsin at 37°C for four hours. Biological triplicates were prepared for each sample type: light HeLa/CDDP to heavy HeLa, heavy HeLa/CDDP to light HeLa, light HeLa/CDDP to heavy HeLa/CDDP, and light HeLa to heavy HeLa. Samples were stored at −80°C until analyzed by LC-MS.

### Mass Spectrometric Analysis

Peptide samples were loaded onto a trap column (3 cm×75 µm i.d. packed with Michrom Magic C18AQ 200 Å pore size, 5 µm) and washed for 10 minutes at a flow rate of 2 µL/min with 98% solvent A (H_2_O, 0.1% formic acid) and 2% solvent B (acetonitrile, 0.1% formic acid) using a nanoAcquity UPLC (Waters, Milford MA). Peptides were then fractionated over the analytical column (30 cm×75 µm i.d. packed with Michrom Magic C18AQ 100 Å pore size, 5 µm particles) using a 120 minute linear gradient from 95% solvent A, 5% solvent B to 60% solvent A, 40% solvent B at a flow rate of 300 nL/min. Peptides were ionized by electrospray ionization (ESI) using a spray voltage of 2.0 kV. Data dependent mass spectrometric analysis of SILAC samples was performed using a LTQ-Orbitrap mass spectrometer (Thermo). Full MS scans from 400–1400 *m/z* were performed in the Orbitrap mass analyzer with the resolution set to 60,000. Tandem mass spectrometry was performed in the ion trap mass analyzer on the five most abundant precursors detected in the Orbitrap full MS scan. Collision induced dissociation was performed using a normalized collision energy of 35 with a 30 ms activation time and an activation Q of 0.25. Ions selected for MS/MS sequencing were then dynamically excluded from repeated MS/MS events for 60 seconds, using an asymmetric mass window of 0.1 *m/z* on the low side and 1.1 *m/z* on the high side. All samples were analyzed in technical duplicate.

### Data Analysis

MS/MS spectra were converted into MGF peak list files using tools in the Trans-Proteome Pipeline [51] and analyzed using Mascot v. 2.3 (Matrix Sciences) database searching software [52]. The spectra were searched against the UniProt database (07/07/10) limited to human taxonomy (20,321 sequences; 11,273,645 residues) with the following search parameters: enzyme set to trypsin, 25 ppm precursor ion tolerance, 0.8 Da fragment ion tolerance, carbamidomethylation of cysteine as a fixed modification, ^13^C_6_-lysine and ^13^C_6_^15^N_2_-arginine as variable modifications, and allowing for a single missed cleavage site. The false discovery rate (FDR) was determined by searching the MS/MS spectra against a randomized database. Protein identifications were made for the highest scoring protein matches to the identified peptides from Mascot.

Peptide sequences identified with Mascot were exported as comma separated value (.csv) files using significance and expect value thresholds of 0.05. This significance level was chosen to give a FDR of less than 1%. Precursor mass spectra were processed using Hardklör [53] to identify peptide isotope distributions and chromatographic elution profiles. The Hardklör results and the .csv files were then analyzed with an in-house developed program, SILACtor, to label the precursor ions with peptide sequences and determine the integrated peak areas for each SILAC pair of isotope distributions. SILACtor provides several useful features not currently available in other SILAC data analysis software including; the generation of accurate mass and time target lists for targeted MS/MS analysis of peptides not identified by the initial analysis, and the ability to trace and compare SILAC labeled peptides across multiple time points. The details of SILACtor will be described in a forthcoming publication, however in our experience SILACtor demonstrates similar quantitative performance as MaxQuant [54] as shown in figure S3. SILAC isotope pairs with mass differences of 6.0201324, 8.0142036, 12.0402648, 14.034336, and 16.0284072 Da were considered, allowing for the possibility of a single missed tryptic cleavage site within a peptide sequence which results in the presence of two isotopically labeled Lys or Arg residues. An accurate mass and retention time database was constructed consisting of the Mascot identified peptide sequences with their respective SILAC ratios. Peptides which were not present in at least 25% of the samples, had retention times which varied for more than 10 minutes, or were identified in the wash or equilibration phases of the chromatography were excluded from the database. Results from technical replicates for each sample were combined by matching SILAC pairs with accurate mass (5 ppm) and retention time (5 minutes) and averaging the SILAC ratios across the replicate analyses. Protein SILAC ratios were obtained by averaging their constituent peptide SILAC ratios. Average protein SILAC ratios were obtained by averaging the protein SILAC ratios from biological triplicates. Peptides found with SILAC ratios that exceeded twice the standard deviation derived from measurements on other peptides from the same protein were removed from the protein analysis and the average protein RIA was re-calculated. The SILAC RIA values were normalized so that the mean value for a given sample was 0.5 under the assumption that the majority of proteins on average will have a 1∶1 ratio between the samples. To determine which proteins had significantly shifted SILAC ratios between the samples consisting of mixed HeLa/CDDP and HeLa proteins versus the same cell type mixed with itself a one way ANOVA was performed using the statistical computing package R. ANOVA was limited to proteins which were observed in at least half of the HeLa/CDDP samples and half of the one to one mixtures of the same cell type samples. Ratios were considered significantly different if they had a p-value of less than 0.01. To generate an expression level heat map, SILAC ratios were analyzed by hierarchical clustering using Gene Cluster 3.0 and visualized using TreeView [55]. A protein interaction network for the 856 proteins identified was constructed with STRING 8 [56]. The protein interaction network generated from the STRING database was visualized with Cytoscape [57]. To obtain enriched GO biological pathway and molecular function terms related to cisplatin resistance, BiNGO [14] was used to analyze the proteins with SILAC ratios that were determined to be significantly increased or decreased (ANOVA, p<0.01) by at least 1.25 fold in HeLa/CDDP compared with HeLa. Settings for BiNGO included using a hypergeometric test with a significance threshold of 0.05. The Benjamini & Hochberg false discovery rate correction was applied to the resulting p-values. Proteins were compared against the full human annotation GO database.

### Western Blotting

Cell lysate samples from HeLa and HeLa/CDDP containing 20 µg of total protein were separated by one dimensional SDS-PAGE using a constant voltage of 100 V. Proteins were transferred to nitrocellulose membranes using 150 mA constant current for 2 hrs. Membranes were blocked by incubation for one hour in PBST (PBS containing 0.1% tween 20) and 5% w/v milk. Membranes were washed with PBST then incubated for two hours at room temperature with a 1∶1000 dilution of primary antibody (CD44, DDB-1, DJ-1, or Ku80) and a 1∶5000 dilution of anti-β-actin antibody in PBST containing 5% w/v BSA. Following extensive washing the membranes were incubated with a 1∶20,000 dilution of goat anti-rabbit IRDye 680 and goat anti-mouse IRDye 800 antibodies. Western blots were imaged with a LI-COR Odyssey infrared imaging system.

## Supporting Information

Figure S1 Full heat map image with dendrogram generated from SILAC ratios for 856 proteins. In total there are 12 lanes representing 3 biological replicates from 4 sample types. Lanes 1–3 are light HeLa/CDDP, heavy HeLa. Lanes 4–6 are heavy HeLa/CDDP, light HeLa. Lanes 7–9 are a light and heavy mixture of HeLa/CDDP. Lanes 10–12 are a light and heavy mixture of HeLa. The averaged version of this heat map is shown as **Figure 3** in the main text for clarity, however the full version allows for the visualization of the biological replicates.
(PDF)

Figure S2 Western Blot images for four proteins that were identified by SILAC to have increased levels in cisplatin resistant HeLa cells. Lanes 1–3 are samples from normal HeLa while lanes 4–6 are from HeLa/CDDP. In each blot β-actin was monitored as a quantitative control and is visible as a green band at 40 kDa. **A** CD44 Western blot with CD44 visible as a green band at 80 kDa. The averaged ratio of the signal intensity from HeLa/CDDP to HeLa was 1.31 prior to normalization and 2.21 after normalization to the signal intensity from β-actin. **B** DDB1 Western blot with DBB1 visible as a red band at 120 kDa. The averaged ratio of the signal intensity from HeLa/CDDP to HeLa was 1.46 prior to normalization and 1.13 after normalization to the signal intensity from β-actin. **C** DJ-1 Western blot with DJ-1 visible as a red band at 22 kDa. The averaged ratio of the signal intensity from HeLa/CDDP to HeLa was 2.75 prior to normalization and 3.28 after normalization to the signal intensity from β-actin. **D** XRCC5 (Ku80) Western blot with Ku80 visible as a red band at 85 kDa. The averaged ratio of the signal intensity from HeLa/CDDP to HeLa was 1.21 prior to normalization and 1.11 after normalization to the signal intensity from β-actin.
(PDF)

Figure S3 Distribution of RIA values for a set of quantified SILAC peptide pairs from a single control sample, comparing the quantification results from SILACtor with MaxQuant.
(PDF)

Table S1 Table of 856 proteins identified and quantified.
(XLS)

Table S2 Enriched Gene Ontology Biological Pathway and Molecular Function Terms for Differentially Expressed Proteins.
(XLS)

Dataset S1 Interactive Cytoscape network.
(TAR)
