# Supplementary material for: Quantitative Proteomic and Interaction Network Analysis of Cisplatin Resistance in HeLa Cells
Source: PLoS One. 2011 May 26;6(5):e19892. doi: 10.1371/journal.pone.0019892 (PMC3102677; doi:10.1371/journal.pone.0019892)
Supplement: Figure S1 — Full heat map image with dendrogram generated from SILAC ratios for 856 proteins. In total there are 12 lanes representing 3 biological replicates from 4 sample types. Lanes 1–3 are light HeLa/CDDP, heavy HeLa. Lanes 4–6 are heavy HeLa/CDDP, light HeLa. Lanes 7–9 are a light and heavy mixture of HeLa/CDDP. Lanes 10–12 are a light and heavy mixture of HeLa. The averaged version of this heat map is shown as Figure 3 in the main text for clarity, however the full version allows for the visualization of the biological replicates. (PDF) [file pone.0019892.s001.pdf]

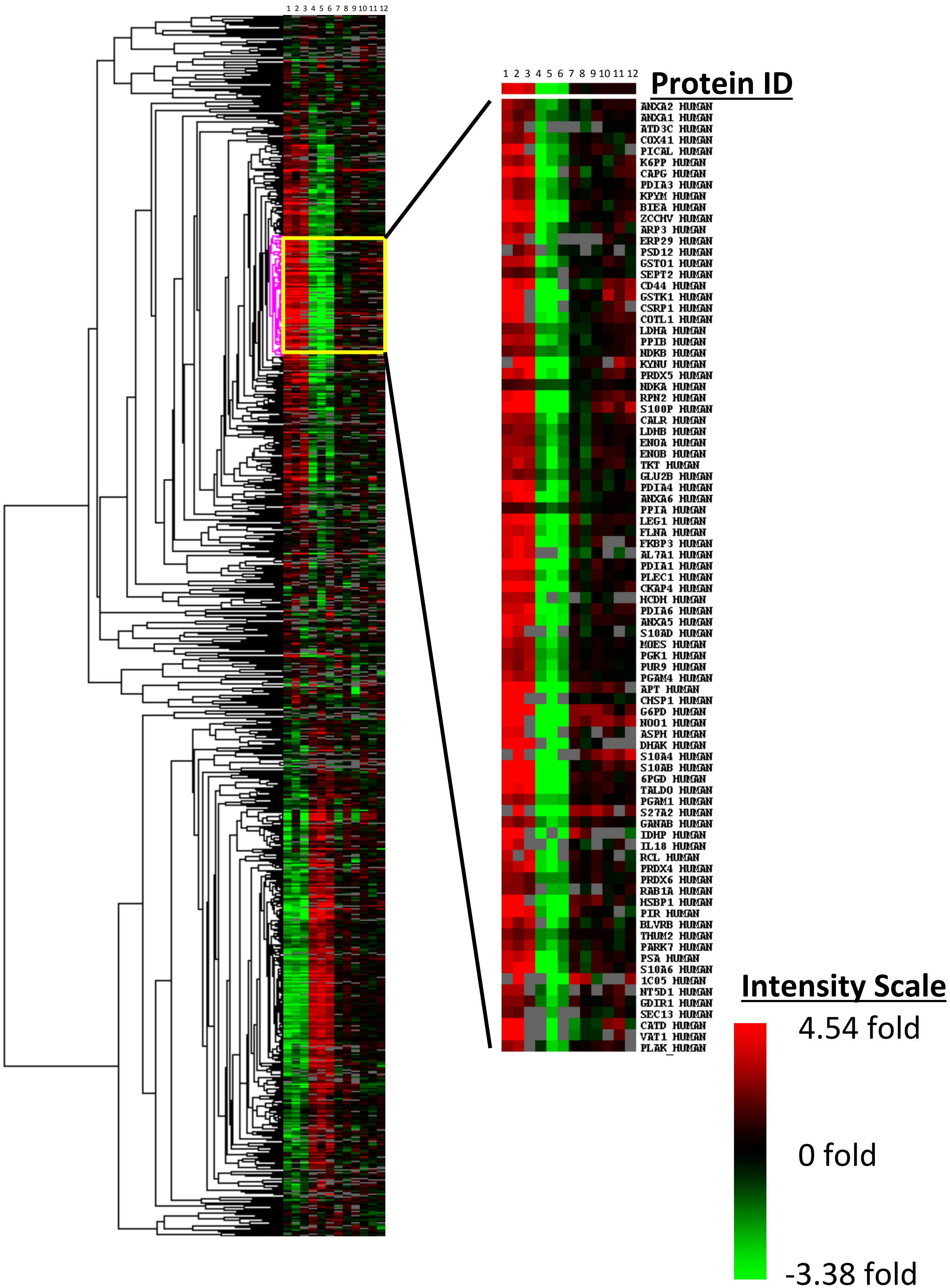
1 2 3 4 5 6 7 8 9 10 11 12
1 2 3 4 5 6 7 8 9 10 11 12
Protein ID
ANXA2 HUMAN
ANXA1 HUMAN
ATD3C HUMAN
COX41 HUMAN
PICAL HUMAN
K6PP HUMAN
CAPG HUMAN
PDIA3 HUMAN
KPYM HUMAN
BIEA HUMAN
ZCCHV HUMAN
ARP3 HUMAN
ERP29 HUMAN
PSD12 HUMAN
GSTO1 HUMAN
SEPT2 HUMAN
CD44 HUMAN
GSTK1 HUMAN
CSRP1 HUMAN
COTL1 HUMAN
LDHA HUMAN
PPIB HUMAN
NDKB HUMAN
KYNU HUMAN
PRDX5 HUMAN
NDKA HUMAN
RPN2 HUMAN
S100P HUMAN
CALR HUMAN
LDHB HUMAN
ENOA HUMAN
ENOB HUMAN
TKT HUMAN
GLU2B HUMAN
PDIA4 HUMAN
ANXA6 HUMAN
PPIA HUMAN
LEG1 HUMAN
FLNA HUMAN
FKBP3 HUMAN
AL7A1 HUMAN
PDIA1 HUMAN
PLEC1 HUMAN
CKAP4 HUMAN
HCDH HUMAN
PDIA6 HUMAN
ANXA5 HUMAN
S10AD HUMAN
MOES HUMAN
PGK1 HUMAN
PUR9 HUMAN
PGAM4 HUMAN
APT HUMAN
CHSP1 HUMAN
G6PD HUMAN
NQO1 HUMAN
ASPH HUMAN
DHAK HUMAN
S10A4 HUMAN
S10AB HUMAN
6PGD HUMAN
TALDO HUMAN
PGAM1 HUMAN
S27A2 HUMAN
GANAB HUMAN
IDHP HUMAN
IL18 HUMAN
RCL HUMAN
PRDX4 HUMAN
PRDX6 HUMAN
RAB1A HUMAN
HSBP1 HUMAN
PIR HUMAN
BLVRB HUMAN
THUM2 HUMAN
PARK7 HUMAN
PSA HUMAN
S10A6 HUMAN
1C05 HUMAN
NT5D1 HUMAN
GDIR1 HUMAN
SEC13 HUMAN
CATD HUMAN
VAT1 HUMAN
PLAK_HUMAN
Intensity Scale
4.54 fold
0 fold
-3.38 fold
