# Supplementary material for: Quantitative Proteomic and Interaction Network Analysis of Cisplatin Resistance in HeLa Cells
Source: PLoS One. 2011 May 26;6(5):e19892. doi: 10.1371/journal.pone.0019892 (PMC3102677; doi:10.1371/journal.pone.0019892)
Supplement: Figure S2 — Western Blot images for four proteins that were identified by SILAC to have increased levels in cisplatin resistant HeLa cells. Lanes 1–3 are samples from normal HeLa while lanes 4–6 are from HeLa/CDDP. In each blot β-actin was monitored as a quantitative control and is visible as a green band at 40 kDa. A CD44 Western blot with CD44 visible as a green band at 80 kDa. The averaged ratio of the signal intensity from HeLa/CDDP to HeLa was 1.31 prior to normalization and 2.21 after normalization to the signal intensity from β-actin. B DDB1 Western blot with DBB1 visible as a red band at 120 kDa. The averaged ratio of the signal intensity from HeLa/CDDP to HeLa was 1.46 prior to normalization and 1.13 after normalization to the signal intensity from β-actin. C DJ-1 Western blot with DJ-1 visible as a red band at 22 kDa. The averaged ratio of the signal intensity from HeLa/CDDP to HeLa was 2.75 prior to normalization and 3.28 after normalization to the signal intensity from β-actin. D XRCC5 (Ku80) Western blot with Ku80 visible as a red band at 85 kDa. The averaged ratio of the signal intensity from HeLa/CDDP to HeLa was 1.21 prior to normalization and 1.11 after normalization to the signal intensity from β-actin. (PDF) [file pone.0019892.s002.pdf]

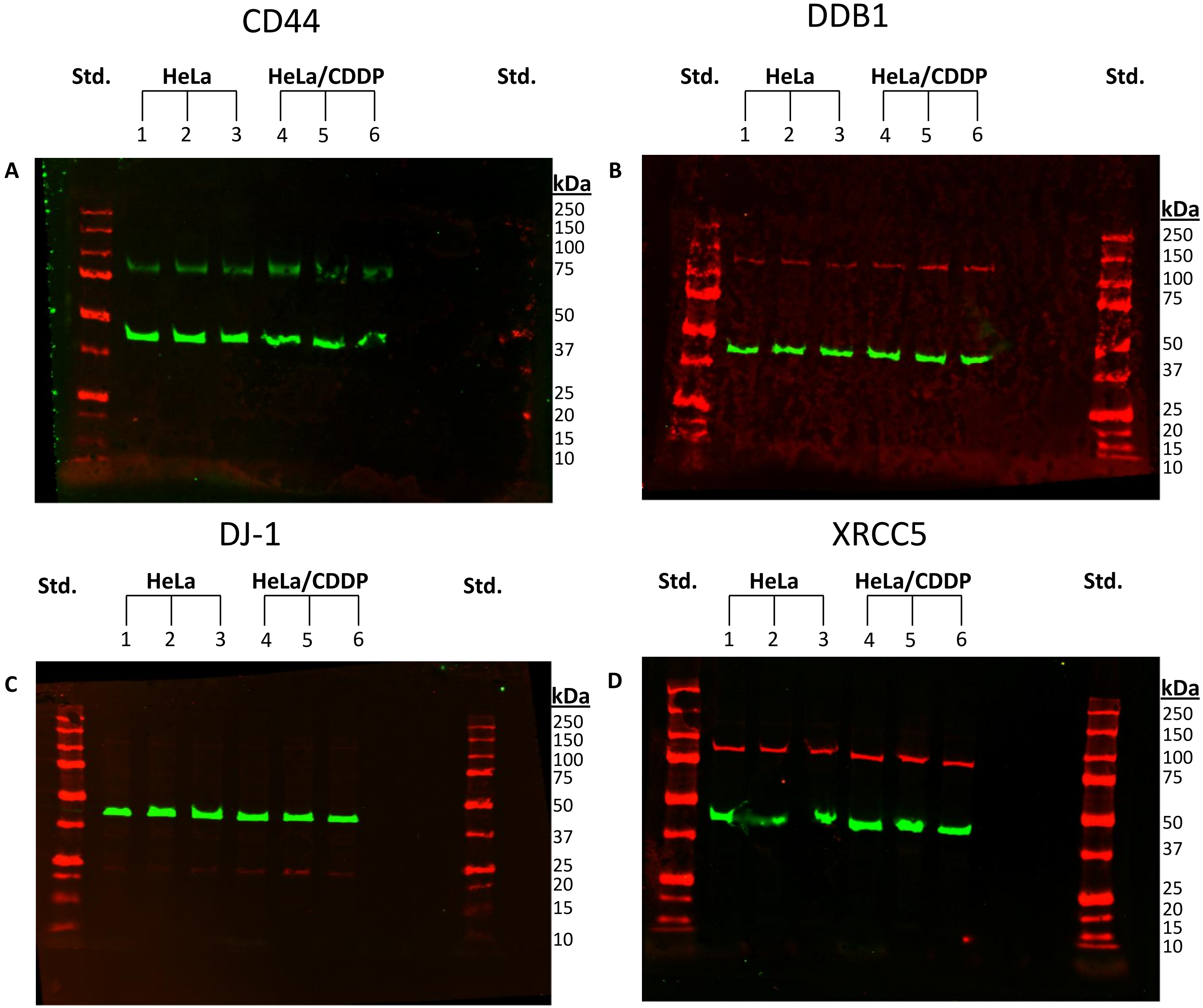

CD44
Std.
HeLa
HeLa/CDDP
Std.
1 2 3 4 5 6
A
kDa
250
150
100
75
50
37
25
20
15
10
DDB1
Std.
HeLa
HeLa/CDDP
Std.
1 2 3 4 5 6
B
kDa
250
150
100
75
50
37
25
20
15
10
DJ-1
Std.
HeLa
HeLa/CDDP
Std.
1 2 3 4 5 6
C
kDa
250
150
100
75
50
37
25
20
15
10
XRCC5
Std.
HeLa
HeLa/CDDP
Std.
1 2 3 4 5 6
D
kDa
250
150
100
75
50
37
25
20
15
10
