# Supplementary figures and images for: Quantitative Proteomic and Interaction Network Analysis of Cisplatin Resistance in HeLa Cells
Source: PLoS One. 2011 May 26;6(5):e19892. doi: 10.1371/journal.pone.0019892 (PMC3102677; doi:10.1371/journal.pone.0019892)

# MaxQuant vs. SILACtor

## Distribution of Normalized RIA values

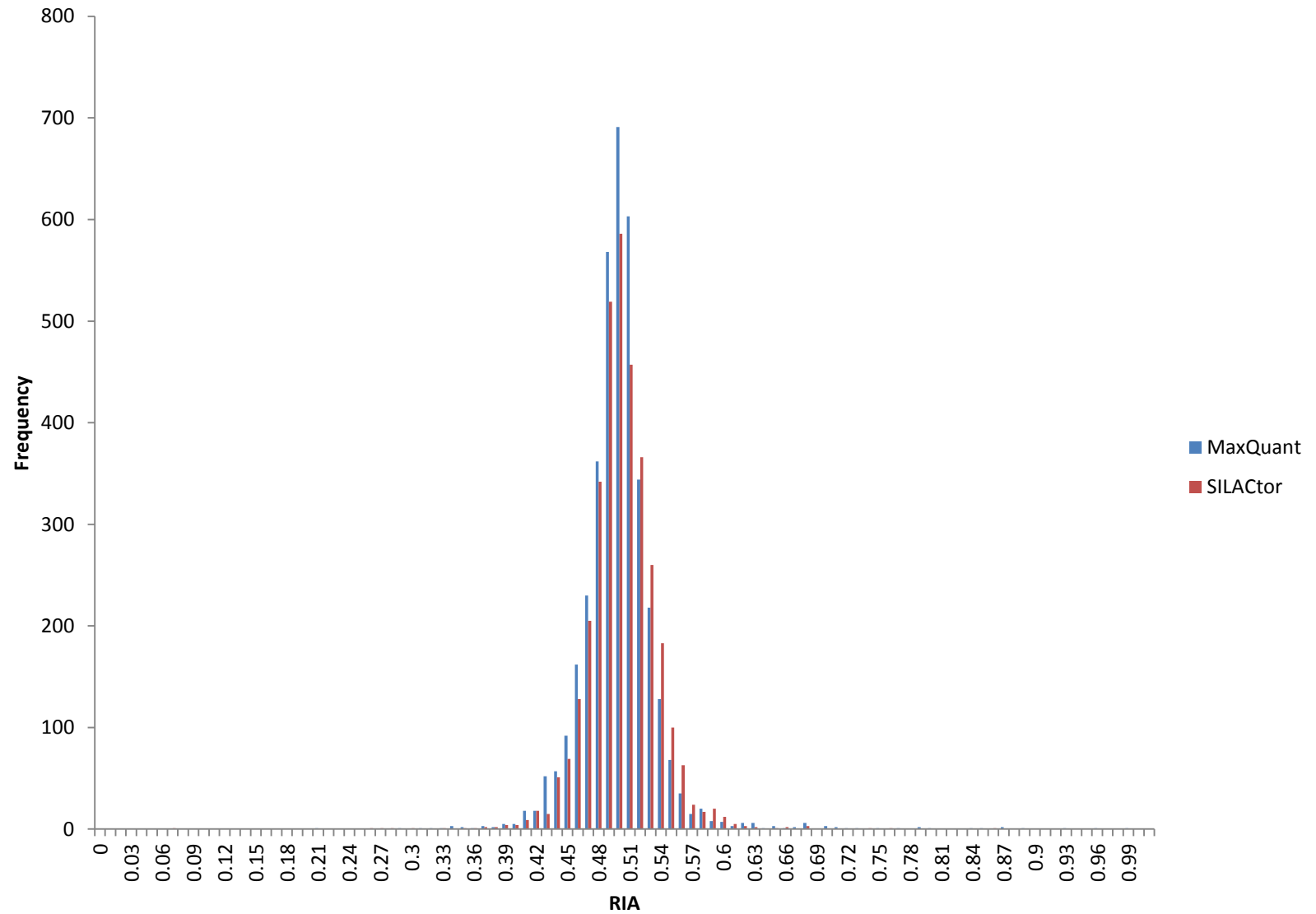

Supplement: Figure S3 — Distribution of RIA values for a set of quantified SILAC peptide pairs from a single control sample, comparing the quantification results from SILACtor with MaxQuant. (PDF) [file pone.0019892.s003.pdf]
